# Annotations

**Published:** 1899-06-24

**Authors:** 


### Annotations.

### Consciousness and Anæsthetics.

A curious article in the *British Medical Journal*—an article suggestive of many doubts rather than indicative of any answer thereto—throws out the question whether an anæsthetic really does produce unconsciousness, or whether it does not rather cause a loss of memory, a state not of unconsciousness but of oblivion. There are two sets of facts which suggest that, however little we may remember what has happened, consciousness is not entirely abolished even while fully under the influence of an anæsthetic. One of these has to do with the horrible feelings with which some people maintain they have been oppressed while under ether or chloroform. This, however, is a bit of evidence to which we need hardly attribute much importance. These feelings are probably "relics of the transition stage, either when passing into or when emerging from anæsthesia;" and, as in dreams, what seems to have been the experience of an hour may probably have been the outcome of a sensation lasting not more than half a minute or even less. Other evidence as to the retention of consciousness is derived from the undoubted retention of all the signs of consciousness, as shown by gestures and cries, which is by no means uncommon during anæsthesia. Now we shall probably be perfectly right in saying that such signs of consciousness need not imply the action of the highest centres, being probably of the nature of reflex actions carried out on a much lower plane, and that their presence is no proof that "we" feel. But so much depends on how we define consciousness! To say that one's consciousness is the sum of one's memories suggests a path out of the difficulty, and should make one quite easy about the signs of suffering exhibited during anæsthesia—for if not remembered they do not matter. During anæsthesia the shutters are up, the office is closed, and the pigeon-holes in which memory is stored away are inaccessible to sensation and to pain. A simple conception enough; but probably far too simple to be true.

### The London Polyclinic.

The highly successful inaugural dinner of the London Medical Graduates' College and Polyclinic, which took place last week, set the seal of permanence upon an institution the possibility of establishing which upon a firm and stable footing many have doubted, even while admitting its desirability and its necessity. The difficulties which have had to be overcome have been manifold, partly in consequence of the enormous field of knowledge with which medical science is in touch, partly because of the very varied wants of those for whose benefit the college is instituted, and partly from the fact that its clientele must always be drawn from the less wealthy members of the profession. Medical men, as a rule, are not rich unless successful, and when success has come the time for college work has mostly passed away; thus the money difficulty has from the beginning stared the college in the face. Not that there need be any doubt of its obtaining a good and satisfactory income from the fees of its students when once it has become established, but there always has been a doubt whether any such institution, unless liberally and influentially supported at the beginning, could keep upon its feet until the arrival of the time when the advantages to be derived from connection with it should have become apparent to all men, and the habit of attending its classes and its consultations from time to time should have eaten its way into the life of the better sort among the younger generation of medical men. This doubt has now been solved by the success of the dinner and the list of the subscriptions to the funds which was there announced, and we may add by the enthusiasm and liberality of Mr. Jonathan Hutchinson. Four thousand guineas is a good round sum; and, although it goes but a little way in the establishment of such a fine and well equipped institution as the Polyclinic already is, even in these early days, it is at least such a sum as will ensure that the scheme will have a full and honest trial and an opportunity for achieving permanent success. This is something, and we shall be greatly disappointed if the undertaking does not have the effect of drawing to London a very large number of earnest post-graduate students who at present pass by on the other side, seeking refuge in Berlin, Vienna, and, to our shame be it said, even in the smaller German Universities, as well as of providing for resident practitioners a clinical college by aid of which, much more easily and more fully than by mere reading, they may keep in touch with all that is newest and most useful in medical science.

### The Right Kind of Lamps.

If we cannot get good oil it is the more necessary that we should have good lamps. Any regulations, however, that may be made by statute with this object can affect only the lamps made in the future, not those in use at present, unless, indeed, an anxious Government will give us new lamps for old. But it is a good thing in any case to know what is the best lamp to use for safety. Dr. Stevenson Macadam, lecturer in chemistry at the Edinburgh College of Surgeons, has published a pamphlet which incidentally touches on the question of lamps. Contrary, as it might seem, to expectation, Dr. Macadam prefers one with a glass fount, in which you can see how the oil is diminishing. He objects to founts which have any special opening for the purpose of filling. He protests, too, against the use of the popular standard lamps. "Many of the founts in these lamps become very highly heated from imperfectly aired burners and from the large shades which are placed above them, and which are often heavily ornamented with coloured muslin or tissue paper of the flimsiest and most inflammable nature. With any oil, but more especially with the low-flash oils, these standard lamps are most dangerous, not only from the possibility of explosion within the lamp fount, but from the facility with which such a tall lamp can be upset by a push, or by an article falling against it. . . . These standard lamps are most unsafe in houses, and should only be tolerated when the base of each is sufficiently weighted or is securely bolted to the floor or wall, and when the top-heavy combustible shade is discarded." The standard lamp is so precious to the soul of the would-be æsthetic house-mistress, and the shade to the bazaar worker, that we fear it may be difficult to get rid of them. But it is well to recognise the dangers attendant on their use, so that, even if they are not totally abolished, they may be guarded with the utmost care. After all, they are not much of a success as illuminating agents. Would it not be quite as satisfactory to keep them purely for ornament, with no oil in the founts, and never try to light them?

